# Optimal likelihood-ratio multiple testing with application to Alzheimer’s disease and questionable dementia

**DOI:** 10.1186/1471-2288-15-9

**Published:** 2015-01-30

**Authors:** Donghwan Lee, Hyejin Kang, Eunkyung Kim, Hyekyoung Lee, Heejung Kim, Yu Kyeong Kim, Youngjo Lee, Dong Soo Lee

**Affiliations:** Department of Statistics, Ewha Womans University, Seoul, Korea; Department of Nuclear Medicine, Seoul National University College of Medicine, Seoul, Korea; Data Science for Knowledge Creation Research Center, Seoul National University, Seoul, Korea; Interdisciplinary Program in Cognitive Science, Seoul National University, Seoul, Korea; Department of Nuclear Medicine, Seoul Metropolitan Government Seoul National University Boramae Medical Center, Seoul, Korea; Department of Statistics, Seoul National University, Seoul, Korea; Department of Molecular Medicine and Biopharmaceutical Sciences, Graduate School of Convergence Science and Technology, and College of Medicine, Seoul National University, Seoul, Korea

## Abstract

**Background:**

Controlling the false discovery rate is important when testing multiple hypotheses. To enhance the detection capability of a false discovery rate control test, we applied the likelihood ratio-based multiple testing method in neuroimage data and compared the performance with the existing methods.

**Methods:**

We analysed the performance of the likelihood ratio-based false discovery rate method using simulation data generated under independent assumption, and positron emission tomography data of Alzheimer’s disease and questionable dementia. We investigated how well the method detects extensive hypometabolic regions and compared the results to those of the conventional Benjamini Hochberg-false discovery rate method.

**Results:**

Our findings show that the likelihood ratio-based false discovery rate method can control the false discovery rate, giving the smallest false non-discovery rate (for a one-sided test) or the smallest expected number of false assignments (for a two-sided test). Even though we assumed independence among voxels, the likelihood ratio-based false discovery rate method detected more extensive hypometabolic regions in 22 patients with Alzheimer’s disease, as compared to the 44 normal controls, than did the Benjamini Hochberg-false discovery rate method. The contingency and distribution patterns were consistent with those of previous studies. In 24 questionable dementia patients, the proposed likelihood ratio-based false discovery rate method was able to detect hypometabolism in the medial temporal region.

**Conclusions:**

This study showed that the proposed likelihood ratio-based false discovery rate method efficiently identifies extensive hypometabolic regions owing to its increased detection capability and ability to control the false discovery rate.

## Background

Several multiple hypothesis testing methods have been proposed for use in neuroimaging studies. Bonferroni correction is the simplest but the most conservative method for controlling the family-wise error rate (FWER). However, it often fails to detect voxels with real activation or difference. As an alternative approach, the Benjamini and Hochberg [1] method for controlling the false discovery rate (FDR) was applied to neuroimaging studies by Genovese, Lazar and Nichols [2]. The FDR control gives statistically less conservative procedures than FWER. However, Cohen and Sackrowitz [3] proved that the Benjamini and Hochberg procedure is inadmissible under any loss function that is a linear combination of false discoveries and false non-discoveries. Given a fixed FDR, it is desirable to maximize the power by minimizing the false non-discovery rate (FNDR).

Recently, Lee and Bjørnstad [4] proposed a new multiple hypothesis test based on the likelihood-ratio-based FDR (LR-FDR). They showed that the problem of large-scale multiple testing is naturally expressed as an inference problem for finding the true discoveries. And they represented the underlying effects of interest by the (unknown) discrete random variables. Statistical inferences are for two types of unknowns, namely parameters (fixed unknowns) and unobservables (random unknowns). Bjørnstad [5] showed that all information on parameter and unobservable data was in the extended likelihood, such as the h-likelihood [6]. Lee, Nelder and Pawitan [7] extensively introduced a random effect analysis using the extended likelihood. More recently, Lee and Bjørnstad [4] showed how the extended likelihood can be used to derive their proposed LR-FDR method. This method is optimal when (a) determining the order in which the test results can be called significant and (b) controlling error rates given this order. Provided an assumed statistical model is true, the likelihood exploits all information in the data to provide the most efficient testing. Therefore, it is important to search for the best-fitting model enhancing the performance of a multiple hypothesis test. The likelihood approach provides various well-developed model-checking and model-selection procedures.

In reviewing existing multiple tests, Efron [8] began by summarizing statistics such as *p*-values [1] and test statistics [8]. He then described how to find a single-threshold rule for such statistics by assuming a common alternative. A typical analysis process is involved in model selection and model prediction. Model selection aims to find a parsimonious, well-fitting model for the basic responses and model prediction uses summarizing statistics from the primary analysis to make statistical inferences [9]. However, starting with the summarizing statistics makes the model selection for the basic responses secondary and difficult, leading to inefficient tests [4]. In addition, assumptions about a common alternative may not always be feasible. For BH-FDR, the conventional t-statistics (and corresponding p-value) are used for testing the difference of means between two groups. The LR-FDR method models with the basic response, not summarizing statistics, which allows for different alternatives for each test. The likelihood approach provides an efficient way of controlling the FDR by simultaneously minimizing the FNDR and the useful information such as consistent estimates of effect size or proportion of null hypotheses.

In this study, we first applied the LR-FDR method to simulated data with extensive alternative proportion (hypometabolic areas in neuroimaging data) and then to brain positron emission tomography (PET) data of three groups: Alzheimer’s disease (AD), questionable dementia (QD), and normal controls (NC). QD is also known as mild cognitive impairment (MCI), and the QD patients in our study were particularly at risk of developing dementia in the near future. We extended the model of Lee and Bjørnstad [4] to allow the distribution of test-statistic is asymmetric.

We compared the LR-FDR method to conventional thresholding using Benjamini and Hochberg’s FDR (BH-FDR), to establish its efficiency when determining hypometabolic regions in AD and QD groups.

## Methods

### The LR-FDR method

Consider a hierarchical model for the basic responses. For the νth location within the brain and the *j*th individual in the control group (*ν =* 1, …, N and *j* = 1,…, *n*_1_), suppose that the response *y*_*vj*1_ is modeled by
1

where *ξ*_*v*_ is the mean parameter and *e*_*vj*1_ ~ *N*(0, *ϕ*_*v*1_). Then, the treatment (or disease) group has *n*_2_ individuals (*j* = 1, …, *n*_2_), and the response *y*_*vj*2_ is modeled by
2

where *w*_*v*_ is the treatment (or disease) effect, and *e*_*vj*2_ ~ *N*(0, *ϕ*_*v*2_). Thus, conditional on *w*_*v*_, the difference between the means of the two groups,
3

follows *N*(*w*_*v*_, *ψ*_*v*_), with *ψ*_*v*_ = *ϕ*_*v*1_/*n*_1_ + *ϕ*_*v*2_/*n*_2_ and , for *v* = 1, …, *N* and *k* = 1, 2. To estimate *ψ* = (*ψ*_1_, …, *ψ*_*N*_), we use the unbiased estimators of *ϕ*_*vk*_ (*v* = 1, …, *N* and *k* = 1, 2),
4

To complete the model, specify the model for the treatment effects, *w*_*v*_.

### One-sided test

Let the null hypothesis *H*_*v*_ be the *v*th voxel is not abnormally activated (not different between two groups). Following Lee and Bjørnstad [4], we defined the binary random variable o_*v*_, such that *o*_*v*_ = 0 if the null hypothesis *H*_*v*_ is true, *o*_*v*_ = 1 if *H*_*v*_ is false, and *p*_*s*_ = *P*(*o*_*v*_ = *s*) for *s* = 0 or 1, with *p*_0_ + *p*_1_ = 1. Now, the multiple test problem can be viewed as predicting *o*_*v*_.

Conditional on *o*_*v*_, assume that *w*_*v*_ follows the normal distribution:


Here, we consider only normal distribution for *w*_*v*_. However, this likelihood approach can be easily extended to other distributions. In this study, we prefer to have *σ*^2^ > 0 since, typically, the null hypotheses “*w*_*v*_ = 0” are never *exactly true*, but rather *w*_*v*_ = 0, which can be modeled by 0 < *Var*(*w*_*v*_) = *σ*^2^. Here, *ψ*_*v*_ in (4) represents the within-test variation, *σ*^2^ the between-test variation for Uninteresting cases, and *τ*^2^ the between-test variation for the Interesting cases. If *ψ*_*v*_ is assumed to be common (i.e., *ψ*_*v*_ = *ψ* for all *v*), this means that there is nothing special about any voxel in the alternative, and they are all statistically exchangeable. How can we determine whether voxels are all (statistically) exchangeable? Since we assume that the *ψ*_*v*_s are not common and estimate them separately, we have a statistical model that allows all active voxels to have the same mean effect, but with different sampling variances. In addition to *ϕ*_*v*1_ and *ϕ*_*v*2_, in this model, we have the fixed parameters *θ* = (*p*_0_, *μ*, *σ*^2^, *τ*^2^).

We denote *d* = (*d*_1_, …, *d*_*N*_)^*T*^ and let *w* and *o* be the vectors of *w*_*v*_ and *o*_*v*_, respectively. In this study, *o* is the inferential focus and *w* is a nuisance parameter which can be integrated out as follows:


where


where *I*(∙) is the indicator function.

To estimate the fixed parameters, *θ*, Lee and Bjørnstad [4] used the maximum-likelihood (ML) estimator for the log-likelihood,
5

where log *f*_*θ*_(*d*_*v*_) = ∑_*ov*_ log *f*_*θ*_(*d*_*v*_, *o*_*v*_) and *ψ*_*v*_ are substituted by  This avoids the downward bias of the ML estimation owing to the large number of nuisance parameters, *ψ*_*v*_, in the model [4].

Since *f*_*θ*_(*d*_*v*_, *o*_*v*_) is a density function for a mixture, the unboundedness of likelihood might occur without a proper constraint on the parameters. However, Hathaway [10] pointed out that this problem can be resolved by a local maximizer of the likelihood in the interior of the parameter space that is consistent and asymptotically efficient. Therefore, to avoid the unboundedness problem, we are actually looking for a good local maximum of the likelihood, which would satisfy both  and [11]. To estimate *θ*, we used the expectation-maximization (EM) algorithm of Dempster, Laird and Rdin [12], with the proper initial values.

Let *δ*_*v*_ be a test for the *v*th null hypothesis, *H*_*v*_ : *δ*_*v*_ = 0 (non-discovery) if *H*_*v*_ is not rejected, and *δ*_*v*_ = 1 (discovery) if *H*_*v*_ is rejected. For some *α* > 0, consider the loss function
6

Lee and Bjørnstad [4] showed that the optimal decision rule, , that minimizes the risk with the loss function (6) is


where  is the likelihood ratio. Among tests with the common expected number of discoveries, this test is optimal in the sense that it controls the FDR with the smallest FNDR.

The outcomes of multiple tests can be summarized as in Table 1. Following Lee and Bjørnstad [4], we define the FDR and FNDR as *E*(*V*)/*E*(*D*) and *E*(*N* − *N*_0_ − *S*)/*E*(*N* − *D*), respectively. Benjamini and Hochberg defined the Fdr as *E*(*V*/*D*), but Genovese and Wasserman [13] showed that Fdr = *E*(*V*/*D*) and FDR = *E*(*V*)/*E*(*D*) are asymptotically equivalent (in *N*) if the tests are independent. Suppose we want a test with an FDR level of *κ*. In this study, we first estimate the FDR as , for each *α*. Then, we search for the cutoff *α* such that  to obtain the optimal test,  with an FDR control of *κ*. Lee and Bjørnstad [4] used the following estimator:

**Table 1:** **The outcomes of N multiple hypothesis tests**

	Non-discovery	Discovery	Total
Null	*N*_0_ − *V*	*V*	*N*_0_
Alternative	*N* − *N*_0_ − *S*	*S*	*N* − *N*_0_
Total	*N* − *D*	*D*	*N*



which works well in their examples from genetic studies in which *N* is of the order of several thousands. However, in brain images for which *N* = 329,694 > > 10,000, we found that *D* can sometimes be less than  To avoid this problem, we use the estimator


In our models, . For a given *α*, the cutoff values  and  can be solved numerically from the equation *R*(*d*_*v*_; *θ*) = *α*. Then,


where Φ(∙) is the cumulative distribution function of a standard normal distribution and  By plugging in the estimates of *θ*, we obtain an estimator for the FDR.

### Two-sided test

In a two-sided problem, we may take only two actions. We can either simply accept or reject the null hypothesis without distinguishing between the positive and negative effects. In the case of brain data, it is important to assign abnormal regional changes at the voxel level. An abnormal voxel can be defined as an abnormally positive (hypermetabolic) or negative (hypometabolic) state. Especially positive activity might be associated with a treatment effect after treatment or functional compensation, while negative activity might be associated with functional deficit. This statistically specific determination of an abnormal voxel influences clinical interpretations. Therefore, this method allows us to consider the sign of the test statistic to decide whether the alternative discovery is positive or negative when the null hypothesis is rejected. In other words, we would never conclude that there is a discovery without stating whether the effect is positive or negative. As we show in the discussion on our results, in neuroimaging, an entire alternative can be either hypermetabolic or hypometabolic, whereas in genetics, alternatives often exist in both directions.

Extending the one-sided test model from the previous section, we used the *two-sided multiple testing with three actions* of Lee and Bjørnstad [4]. Here, the discrete random variable, *o*_*v*_, takes one of the three states: (a) *o*_*v*_ = 0 if the *i*th case is “Uninteresting;” (b) *o*_*v*_ = 1 if the *i*th case is “Interesting, with a Positive effect;” and (c) *o*_*v*_ = − 1 if the *i*th case is “Interesting, with a Negative effect.” In addition, *p*_*s*_ = *P*(*o*_*v*_ = *s*) for s = − 1, 0, 1, with *p*_0_ + *p*_1_ + *p*_− 1_ = 1.

Consider the differences in (3). Suppose that, conditional on *o*_*v*_, *w*_*v*_ follows a normal distribution, as follows:


where *μ*_*P*_, *μ*_*N*_ > 0. For simplicity of arguments, in this paper, we assume that *μ*_*P*_ = *μ*_*N*_ = *μ* and . In this model, we have the fixed parameters *θ* = (*p*_0_, *p*_1_, *μ*, *σ*^2^, *τ*^2^), yielding a three-mixture model. Thus, we can use the EM algorithm to estimate *θ*. Since *o*_*v*_ takes one of three states, the decision rule *δ*_*v*_ also takes a value in {0, 1, − 1}. In other words, *δ*_*v*_ = 0 (non-discovery) if *H*_*v*_ is not rejected, *δ*_*v*_ = 1 if *H*_*v*_ is rejected with a positive effect, and *δ*_*v*_ = − 1 if *H*_*v*_ is rejected with a negative effect.

For some *α*_+_ > 0 and *α*_−_ > 0, consider the loss function
7

Then, the optimal decision rule that minimizes the risk in the loss function (7) is


where


If we control the FDR at level *κ* for both directions using *α*_+_ and *α*_−_, the resulting two-sided test with three actions maintains the FDR at the same level. Furthermore, this optimal test allows more flexible analysis which can control the FDR at different level for each direction, for example, 0.05 for positive direction and 0.01 for negative direction. In fact, Lee and Bjørnstad [4] showed that the resulting multiple two-sided test with three actions  minimizes the expected number of false assignments.

### Simulation data

Simulation data were generated with a dimension of 400 × 400 pixels. We set the proportion of positive pixels to 80% (Simulation I) and 60% (Simulation II) per 160,000 total pixels, considering that the estimates of *p*_1_ were high in our PET data. For each simulation setting, we varied *μ* = 1, 3, 5 and fixed *σ*^2^ = 0.3 and *τ*^2^ = 0.5. From (1) and (2), we randomly generated *y*_*vj*1_ and *y*_*vj*2_ for *v* = 1, …, 160, 000, *j*_1_ = 1, …, 30, and *j*_2_ = 1, …, 30. For each simulation, we generated 100 simulation data sets and applied both the LR-FDR and BH-FDR methods to control the FDR at the 0.05 level.

### AD and QD PET data

PET data were composed of two types of patient groups and one control group. The first group consisted of 22 probable AD patients (mean age, 66.9 ± 7.2) with moderate dementia according to the criteria of the Mini-Mental State Examination (MMSE), with a mean MMSE score of 13 ± 5.0, and a Clinical Dementia Rating (CDR) score between 1 and 3. Generally, the MMSE score can be indicated severe (<9), moderate (10–18), mild (19–24) cognitive impairment. The AD patients suffered progressive memory loss, but had no disturbance of consciousness. The second group comprised 24 QD patients (mean age, 67.3 ± 9.0) who showed objective evidence of memory and/or cognitive impairments, but did not satisfy the criteria for AD. Their CDR scores were all 0.5, and their mean MMSE scores were 23 ± 4.1.

All the patients were diagnosed by clinical evaluation using the National Institute of Neurological and Communicative Disorders and Stroke and Alzheimer’s Disease and Related Disorders Association AD criteria as a guideline. The two patient groups described above were compared with 44 normal control (NC) subjects (mean age, 68.9 ± 5.2). These NC subjects were recruited from the Health Care Center at Seoul National University Hospital and had no history of neurological disorders, psychiatric disorders, significant medical conditions, or substance abuse. For subject screening, the Korean version of the modified MMSE and the Mood Evaluation Scale were used, and only right-handed subjects were included in the study. Furthermore, there was no significant age difference among the three groups. This study was approved by the institutional review board (IRB) of the Seoul National University Hospital. PET data of our patients were only part of the patient’s standard care. We used patient’s data from database obtained from 1996 to 1999. Normal controls were recruited for other study purpose (i.e., creation of Korean Standard Brain Template) from Center for Health Promotion and Optimal Aging of Seoul National University Hospital in 2001 [14], who provided informed consent which was verbal form. For our research using identifiable human data, such as PET data in database of department, although we didn’t receive documented informed consent from participants, IRB of our institute decided that this study protocol was applicable to exceptional situations where consent would be impracticable to obtain due to reuse storage data in database. Also our study was conducted in a manner that minimizes possible abuse to human subject’s health and rights and no clinical intervention was performed for our study.

### PET image acquisition

^18^ F-FDG PET images were obtained using an ECAT EXACT 47 (Siemens-CTI, Knoxville, TN, USA) PET scanner with an intrinsic resolution of 5.2 mm FWHM. After obtaining a transmission scan measured by ^68^Ge rod sources for attenuation correction, an emission scan was obtained. During the resting state, ^18^ F-FDG was administered in doses of 370 MBq (10 mCi) for 30 min to obtain a static emission scan. All participants were scanned under the normal environmental noise conditions in the scanner room. For transaxial image reconstruction a filtered back-projection algorithm (Shepp-Logan filter at a cutoff frequency of 0.3 cycles/pixel as 128 × 128 × 47 matrices of size 2.1 × 2.1 × 3.4 mm) was used.

### Image processing

All PET images were preprocessed using Statistical Parametric Mapping (SPM 2, University College of London, UK) and implemented in the Matlab 6.5 (Mathworks Inc., USA) environment. After spatial normalization to the Montreal Neurological Institute (MNI) space, all images were smoothed with a Gaussian filter of 16 mm full width at half maximum (FWHM). The PET signal intensity was normalized to the individual’s total mean count for the cerebellum. This region was chosen as a reference region because it remains relatively unaffected until late in the progression of AD, if at all. To remove non-brain voxels, normalized and smoothed PET images were exclusively masked with a binary brain mask image. The same masked PET images were applied to both LR-FDR and BH-FDR methods using R software.

## Results

### Simulation results

**Figure 1:**
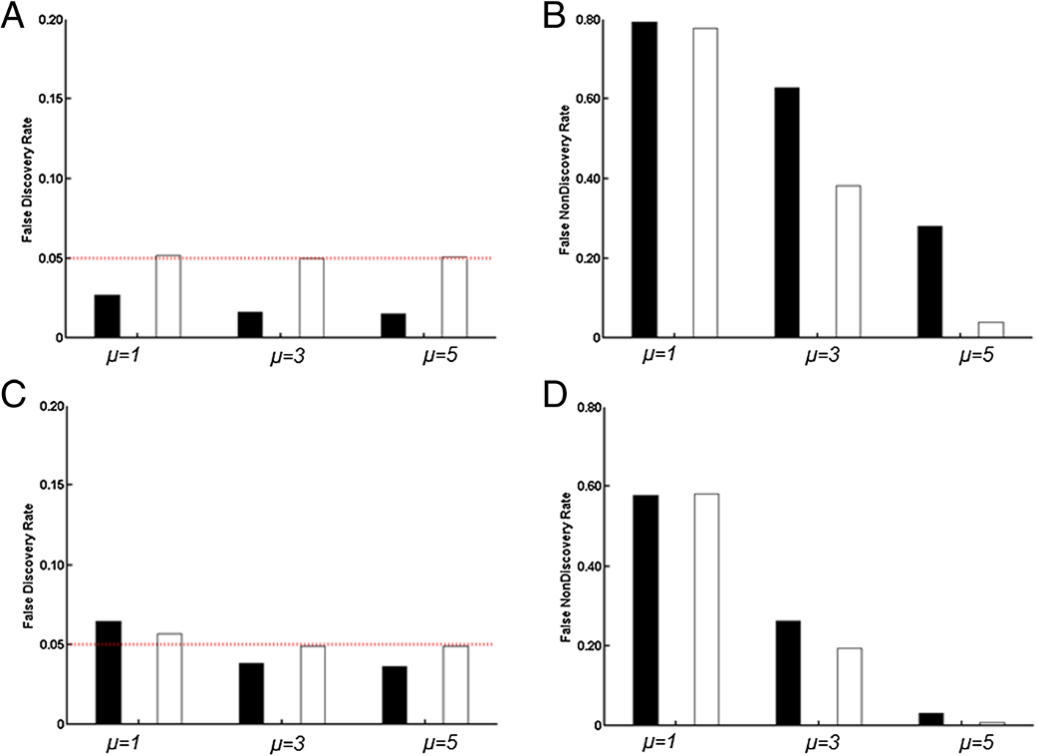
**The averaged FDR and FNDR.** Within each panel, black and white bars represent the BH-FDR and LR-FDR methods, respectively. The alternative proportions of data are 80% and 60% in Simulation I **(A and** **B)** and II **(C and** **D)**, respectively. In each simulation setting, depending on *μ*, three parameter settings are presented

We applied the proposed LR-FDR method to the simulated data set. The simulated data had a pixel dimension of 400 × 400, which yielded a total 160,000 tests. We considered two simulation settings with varying *p*_1_, the proportion of pixels with *o*_*v*_ = 1: *p*_1_ was 80% in Simulation I and 60% in Simulation II. Figure 1 shows the FDR and FNDR results based on the 100 simulated data sets.

The LR-FDR method yielded a smaller FNDR than the BH-FDR method (Figure 1): 20% lower when *μ* = 3 or 5 in Simulation I (*p*_*1*_ = 80%) and 5% lower when *μ* = 3 in Simulation II (*p*_*1*_ = 60%). The LR-FDR method yielded FDR results quite close to 0.05 in both simulation settings. The minimum and maximum of the average FDR from the 100 repeated tests were 0.049 and 0.056, respectively, across all settings. The BH method often yielded a more conservative FDR control for most of the settings.

### Results of the AD and QD data analysis

**Figure 2:**
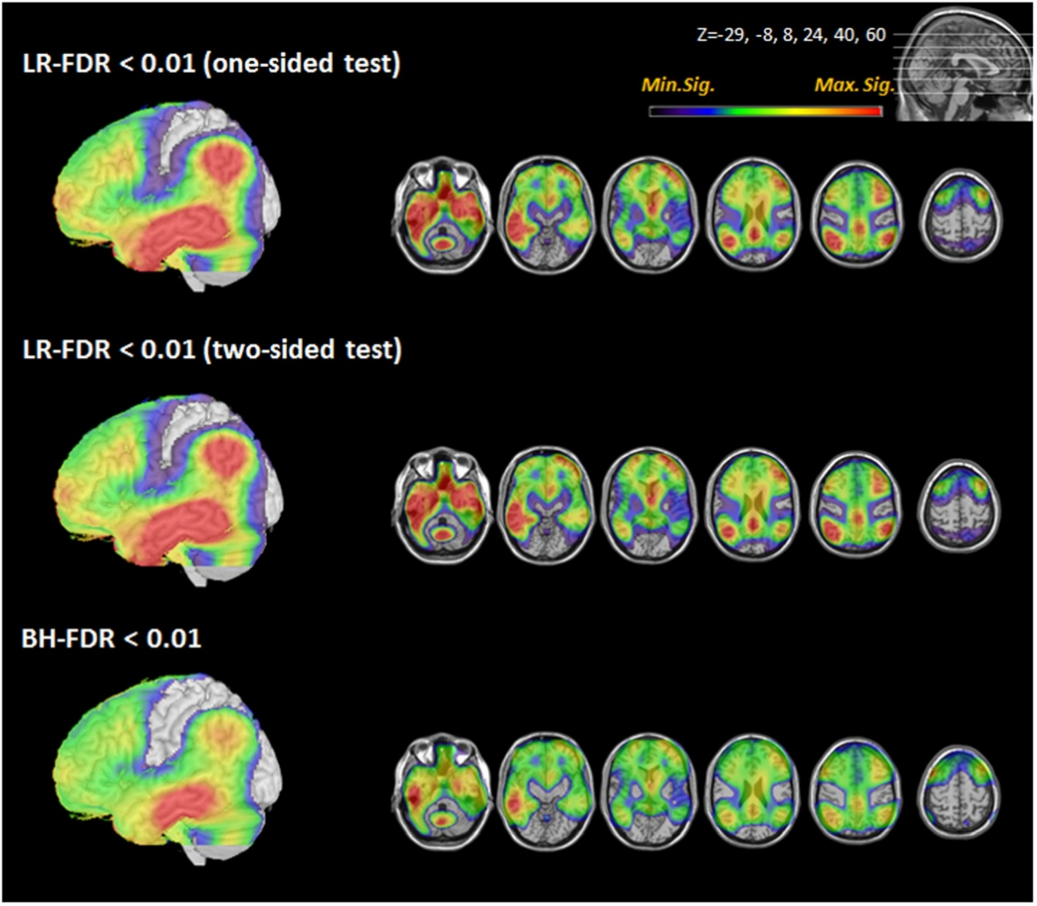
**Brain regions with significantly lower FDG uptake in probable AD compared to NC.** Regions with lower FDG uptake in probable AD are displayed. The left hemisphere is shown as a 3D volume rendering. The reduction in the FDG uptake in the temporal, parietal, and posterior prefrontal regions was commonly found in the LR-FDR and BH-FDR methods. Extensive hypometabolic areas extending to posterior prefrontal were detected with the LR-FDR one-sided and two-sided tests. The color bar range from minimum to maximum significance level denotes the significance of the likelihood ratio in both LR-FDR methods and of the *p*-value in BH-FDR method. (AD: Alzheimer’s disease; FDR: False discovery rate; LR-FDR: Likelihood ratio false discovery rate; NC: Normal controls).

**Figure 3:**
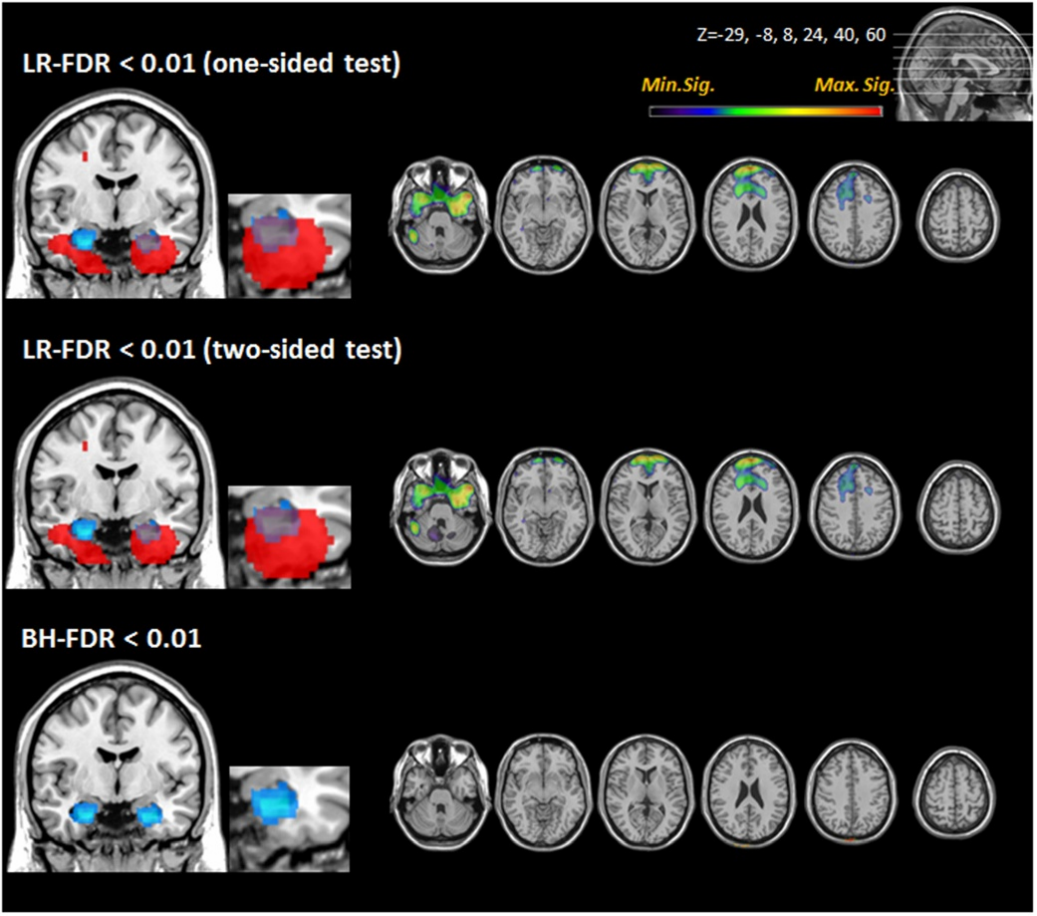
**Brain regions with significantly lower FDG uptake in QD compared to NC.** The coronal view in the left column shows hypometabolism in the medial temporal regions in QD. An anatomical map of the hippocampus is displayed in blue. Regions with a lower FDG uptake are displayed in red. The LR-FDR one-sided and two-sided tests disclosed more extensive hypometabolic areas in both temporal lobes than did the BH-FDR method. (FDR: False discovery rate; LR-FDR: Likelihood ratio false discovery rate; NC: Normal controls; QD: Questionable dementia).

In probable AD cases, all methods (one-sided test of LR-FDR, two-sided test of LR-FDR, conventional BH-FDR methods) revealed hypometabolic regions at FDR level 0.01 (Figure 2). Both the one-sided and two-sided tests of LR-FDR showed hypometabolism in the bilateral posterior cingulate, frontal, temporal, and parietal areas, the extent of which was wider than that shown by conventional BH-FDR. More specifically, the LR-FDR method showed that the hypometabolic regions spread to the posterior prefrontal and anterior occipital regions in the AD group. No hypometabolic areas were observed in the sensorimotor and visual areas by any of the methods. Quantification using the LR-FDR method generally found a greater number of voxels than did the BH-FDR method (Table 2). In the QD cases, the LR-FDR method showed hypometabolic regions in both medial temporal areas, including the hippocampus and anterior frontal cortex (Figure 3). The hypometabolic voxels in the medial temporal regions were found more easily using LR-FDR method at 0.05, 0.01, 0.005, and 0.001. However, no hypometabolic region was found by BH-FDR method with controlling FDR at 0.01 (Table 3).

**Table 2:** **Total number of voxels in the whole brain with significant hypometabolism at different threshold levels**

Comparisons	Threshold levels	Number of significant voxels		
		LR-FDR (one-sided)	LR-FDR (two-sided)	BH-FDR
NC > AD	FDR 0.001	194789	194789	165451
	FDR 0.005	213881	213881	207426
	FDR 0.01	223091	223091	221507
	FDR 0.05	249706	249706	251100
NC > QD	FDR 0.001	8471	7740	47
	FDR 0.005	18094	16767	140
	FDR 0.01	25624	23813	212
	FDR 0.05	53229	50287	22038

AD: Alzheimer’s disease; NC: Normal controls; QD: Questionable dementia.

**Table 3:** **Total number of voxels in hippocampus with significant hypometabolism at different threshold levels**

Comparisons	Threshold levels	Number of significant voxels		
		LR-FDR one-sided (L/R)	LR-FDR two-sided (L/R)	BH-FDR (L/R)
NC > QD	FDR 0.001	0/117	0/117	0/0
	FDR 0.005	1/181	1/184	0/0
	FDR 0.01	12/217	12/217	0/0
	FDR 0.05	118/303	121/303	0/158

NC: Normal controls; QD: Questionable dementia.

The estimates of the fixed parameters are shown in Table 4. In the AD cases, two-sided tests give the effect size,  = 4.524, and the estimated probability of “Interesting, with a Negative effect,”  = 0.771. Since  approaches 0 in the two-sided test, both tests have the same parameter estimates and the same number of significant voxels. In the QD cases, very few hypermetabolic region was found.

**Table 4:** **Parameter estimates**

Comparisons	Tests	***μ***	***σ*** ^2^	***τ*** ^2^	***P***_0_	***P***_1_	***P***_−1_
NC > AD	One-sided	−4.524	0.001	9.437	0.229	0.771	-
	Two-sided	4.524	0.001	9.437	0.229	0.000	0.771
NC > QD	One-sided	−1.437	0.000	0.696	0.669	0.331	-
	Two-sided	1.479	0.000	0.594	0.674	0.004	0.323

AD: Alzheimer’s disease; NC: Normal controls; QD: Questionable dementia.

**Figure 4:**
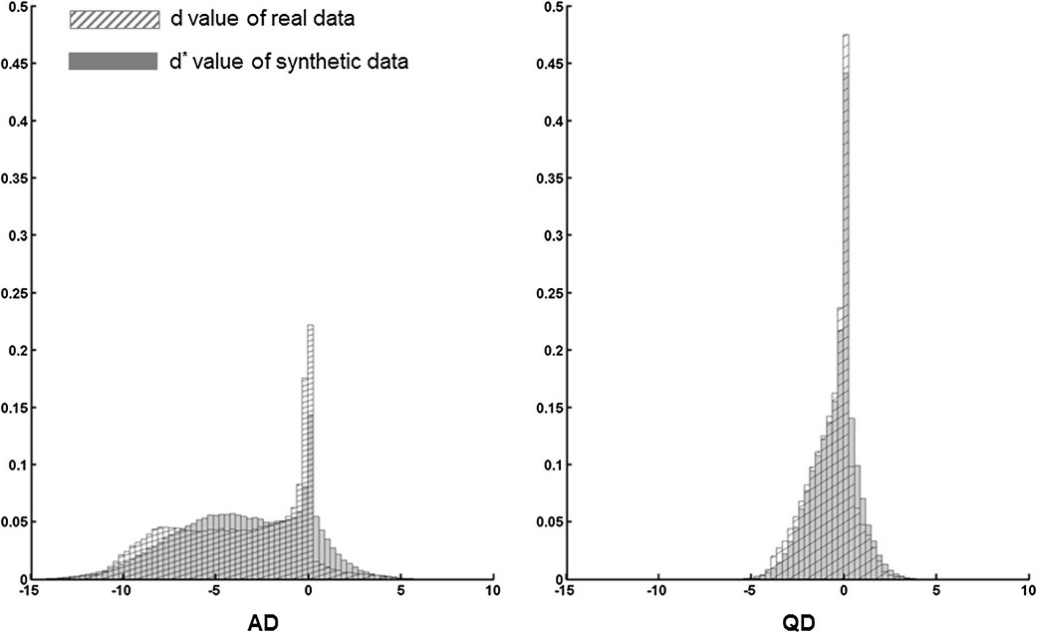
**Histograms of real and synthetic data.** Histograms of  of the generated synthetic data from fitted model (gray histogram) and *d*_*v*_ of real data (hatched histogram) (left: AD cases, right: QD cases) (AD: Alzheimer’s disease; QD: Questionable dementia).

The distribution of  at the null *o*_*v*_ = 0 was *N* (0, 1). However, Figure 4 shows that most *t*_*v*_ -values for both the AD and QD groups were located on the left of the theoretical null distribution, *N* (0, 1). Lee and Bjørnstad [4], when analyzing genetic data, assumed that *p*_1_ = *p*_− 1_, but we did not do so here, as in our neuroimaging data, *p*_1_ ≪ *p*_− 1_. For both the AD and QD PET data, the symmetric model (with *p*_1_ = *p*_− 1_) was not plausible. Therefore, we avoided using the wrong symmetric model by estimating, *p*_1_ and *p*_− 1_ separately. To check the goodness of fit, we first generated a synthetic sample,  from the fitted model, *f*_*θ*_(*d*_*v*_), using the estimated parameters in Table 4. Figure 4 shows the histogram of . Since the shapes of the histograms of the *d*_*v*_ (from the real data) and  (from the synthetic data) were similar, we could say that resulting model fitting was appropriate.

In AD group, the result of the LR-FDR one-sided test was the same as that of the LR-FDR two-sided test with three actions, because in these data, there was no positive effect (). In other words, no hypermetabolic region was found in AD patient group.

## Discussion

In this study, we applied the LR-FDR method to neuroimaging data. We found that the LR-FDR method increased the detection capability in the simulated as well as brain PET data, allowing us to decrease the FNDR and find larger areas of abnormality under the given level of the FDR, respectively. Decreasing the FNDR worked when the difference of the means of the two groups was within a range specified in the simulation study. When we compared the two patient groups (AD and QD) with NC group, the three actions of 1, 0, and −1, corresponding to positive (normal < patients), null (normal = patients), and negative (normal > patients) differences, revealed areas of hyper-, eu-, and hypo-metabolism, respectively. Only negative results (i.e., hypometabolism) in AD patients as compared to normal were obtained and visualized in both the one-sided test and the two-sided test with three actions. In the two-sided test with three actions, the estimated probability of a hypermetabolic region was zero in cases with AD. In these cases, extensive regional metabolic reduction was found throughout the brain, with the same degree, by the one-sided test and two-sided test with three actions.

In existing literature, several reports have stated that abnormalities in glucose metabolism are probably present in the medial part of the temporal lobes early in the development of AD [15, 16]. QD or MCI subjects (CDR score of 0.5) are likely to show initial minor abnormalities [17, 18] that, in some cases, have progressed to probable AD upon follow-up [16, 19, 20]. Various investigators have attempted to find the predictive areas of abnormality, by using both FDG PET [16] and MRI using voxel-based morphometry [21], to predict future development of AD. Among these predictors are medial temporal lobe involvement of MRI signal loss (atrophy) [18–21], accumulation of neutritic plaques [22], and hypometabolism [16, 23].

The expansion of hypometabolism to the temporal, cingulate, or other cortices was a common finding in AD. However, the right/left asymmetry of involvement or the exact nature of the abnormality in the hippocampus shown by FDG PET or MRI has not been consistently reported [18, 24–26]. This might be due to differences between patients and the normal populations examined, but might also be due to differences in the statistical methods used to detect abnormalities. Thus far, considerable effort has been made to control false positives, but less effort has gone into minimizing false negatives. Using this novel LR-FDR method to minimize the FNDR, we found right-dominant abnormalities in the hippocampus in a relatively small patient group.

Using simulation studies, we showed that the LR-FDR method controlled the FDR quite near to the stated level. In contrast, the BH-FDR method did not maintain the stated FDR level, and instead became more conservative (i.e., a lower FDR then set beforehand). Furthermore, the LR-FDR method reduced the FNDR significantly in certain situations, according to the simulation study, as compared to the BH-FDR method. The FNDR reduction became greater as *p*_1_ increased. In the neuroimaging data, such as the AD PET data, *p*_1_ was larger (e.g., 0.771), whereas in the genomic studies, *p*_1_ was often small (i.e., less than 0.05). The BH-FDR method assumes that *σ*^2^ = 0. However, the LR-FDR method allows for non-zero between-test variations (*σ*^2^ > 0 or *τ*^2^ > 0). In our PET study using real imaging data, we found that the maximum likelihood estimates of *τ*^2^ are very different from zero. In a neuroimaging data analysis, the LR-FDR method was preferred over the BH-FDR method. The LR-FDR method had a higher detection capability, and showed extensive hypometabolic regions in patients with AD or QD. Especially, in QD group, no significant area was found in BH-FDR at 0.01, although the hypometabolic voxels were 229 in LR-FDR method. One possibility is that the hippocampal region was falsely assigned as a null in the BH-FDR method at this threshold level.

The data used in this study were drawn from Lee, Kang, Jang, Cho, Kang, Lee, Kang, Lee, Woo and Lee [27], and the assessment of cerebral glucose metabolism by FDG-PET in a resting state correlated well with the progression of disease severity in patients with AD [23, 28]. Unlike patients with cognitive deterioration associated with old age, patients with AD showed decreased FDG uptake in both parietal regions, including the posterior cingulate and temporal areas and the frontal cortices, as the disease progressed [29, 30]. Primary sensory and motor cortices, as well as visual and deep gray cortices remained relatively intact in AD until late in the disease progression [31]. FDG-PET results, analyzed by all three methods, showed a characteristic spatial pattern of glucose hypometabolism in the parietal, temporal, and posterior prefrontal regions in patients with AD, as compared to the NC group. In AD cases, the pattern of distribution was similar. However, unlike the conventional methods, the LR-FDR method showed more extensive hypometabolic areas, extending symmetrically to posterior prefrontal cortices.

Hippocampal atrophy was once thought to be a discriminant feature in individuals with MCI at risk of AD [18, 21]. In our investigation, the LR-FDR method could disclose that reduced FDG uptake in the hippocampal region is a discriminator between normal and QD patients [32, 33]. The BH-FDR method showed no temporal hypometabolic result. In contrast, the LR-FDR method revealed hypometabolism in bilateral medial temporal areas. The hypometabolism seen on the right side was more extensive and severe in LR-FDR method.

We showed that the LR-FDR method for two-sided multiple testing with three actions can be applied to neuroimaging data analysis to find hypermetabolic or hypometabolic regions. In the search for a pre-symptomatic imaging biomarker in the prodromal phase of AD (i.e., QD), we propose that the LR-FDR method is the most efficient tool and, therefore, optimizes the chances for success. According to the good fitting of the model shown in Figure 4, we could say that the non-symmetric model fitting and efficient analysis was feasible to yield robust results from the LR-FDR method, using either the one-sided test or two-sided test with three actions. In the non-symmetric cases, none of the methods employed by Lee and Bjørnstad [4] worked, assuming *p*_1_ = *p*_− 1_. This is the advantage our LR-FDR method holds, when applied to neuroimaging data, over any existing *p*-value based methods.

The extended likelihood principle of Bjørnstad [5] means that if the assumed model is correct, all information on the unknowns is in the extended likelihood. Therefore, this can be the basis for the most efficient test. However, if the assumed model is not correct, the likelihood method may fail. All the existing multiple testing procedures have been developed without considering a proper model choice, so that, as Lee and Bjørnstad [4] showed, existing methods may not maintain the stated FDR level if any of their model assumptions are wrong. Under the likelihood approach, we can use the likelihood-based model-checking and model-selection procedures to enhance the performance of the test [4].

After reviewing the simulation data, we were surprised that the BH-FDR and LR-FDR methods produced so high an FNDR when the difference was small, for example, *μ* = 1. We need to improve the methods to obtain robust results, even when the alternative and null distributions overlap by so much. Another interesting area of future research would be to study robust models for various violations of model assumptions using double hierarchical generalized linear models [7, 34]. Furthermore, the neuroimaging data are actually spatially correlated among the voxels. Owing to the difficulty in specifying the full spatial dependency, we assumed independence over voxels. Genovese, Roeder and Wasserman [35] showed that exploiting the dependency structure improved the power. Thus, a further extension of the LR-FDR method to a spatially correlated model would be a promising prospect for future work.

## Conclusions

We applied the LR-FDR method to PET data from AD and QD patients and compared the performance to that of conventional BH-FDR method. We found that the LR-FDR method enabled us to find more voxels with a congruent distribution. Based on our findings from the AD and QD PET subjects and our simulation study, proving the increased efficiency, bilateral hippocampal hypometabolism might serve as a marker for QD. It would be interesting to extend this approach to perform individual analyses of PET or MRI images to find a meaningful region of brain. A prospective study of a cohort of subjects with QD (or MCI), in which individuals might show a conversion to AD, is warranted, and the LR-FDR method would prove advantageous in such studies.
